# Dataset on the development of palladium nanoparticle decorated colloidal porous organic polymer for photocatalytic Suzuki coupling

**DOI:** 10.1016/j.dib.2018.10.096

**Published:** 2018-10-27

**Authors:** Jeet Chakraborty, Ipsita Nath, Francis Verpoort

**Affiliations:** a Laboratory of Organometallics, Catalysis and Ordered Materials, State Key Laboratory of Advanced Technology for Materials Synthesis and Processing, Center for Chemical and Material Engineering, Wuhan University of Technology, Wuhan 430070, China; b National Research Tomsk Polytechnic University, Lenin Avenue 30, Tomsk 634050, Russia; c Ghent University Global Campus, 119 Songdomunhwa-Ro, Yeonsu-Gu, Songdo, Incheon 406-840, South Korea

## Abstract

Herein, we report the synthesis and characterization data of visible-light-active colloidal azobenzene-based porous organic polymer (Azo–POP) and its Pd-nanoparticle loaded analog (Pd–Azo–POP). The setup for photocatalytic Suzuki reactions triggered by Pd–Azo–POP under conventional batch reaction mode as well as in a prototypal continuous flow system has also been provided in addition to the detailed catalytic data including ^1^H and ^13^C NMR spectra of the obtained products. For further discussions on the materials, their effect on overall catalysis and mechanistic insight, please refer to the associated article “Pd-nanoparticle decorated azobenzene-based colloidal porous organic polymer for visible and natural sunlight-induced Mott–Schottky junction mediated instantaneous Suzuki coupling” (Chakraborty et al., 2019).

**Specifications table**

**Table:** 

Subject area	*Material Science*
More specific subject area	*Mott-Schottky junction mediated porous organic polymer photocatalyst*
Type of data	*Figures, images (microscopy, digital), scheme, tables*
How data was acquired	*Solid-state*^*13*^*C-CP/MAS, FT-IR, BET, FESEM, TEM, Cyclic Voltammogram, and solution state NMR*
Data format	*Analyzed*
Experimental factors	*Azobenzene moieties are incorporated in the porous organic polymer (POP) backbone synthesized by colloidal technique and Pd nanoparticles were loaded on it. The material was used for Mott-Schottky junction mediated visible light-induced photocatalytic Suzuki reactions*
Experimental features	*Catalytic tests performed under visible light and natural sunlight in a batch reactor and in continuous flow method was monitored and analyzed by solution-state NMR*
Data source location	*State Key Laboratory of Advanced Technology for Materials Synthesis and Processing, Center for Chemical and Material Engineering, Wuhan University of Technology, Wuhan, China*
Data accessibility	*Data are accessible with the article*
Related research article	*Pd-nanoparticle decorated azobenzene-based colloidal porous organic polymer for visible and natural sunlight induced Mott-Schottky junction mediated instantaneous Suzuki coupling,* Chemical Eng. J. 358 (2019) 580–588

**Value of the data**

• Visible light and natural sunlight were used as green energy source to furnish Suzuki and Suzuki-type couplings.
• Promoting overall photo-activity of the catalyst through generation of Mott–Schottky heterojunctions at the semiconducting POP–Pd interface after incorporating Pd nanoparticles in the organic network is portrayed.
• The data provide insight into the physical and chemical characteristics of the materials suitable for photocatalytic Suzuki reaction.
• Highest ever TOF in batch mode reaction and first ever instantaneous formation of Suzuki product in a continuous flow reaction setup has been presented.

## 1 Data

**Fig. 1:**
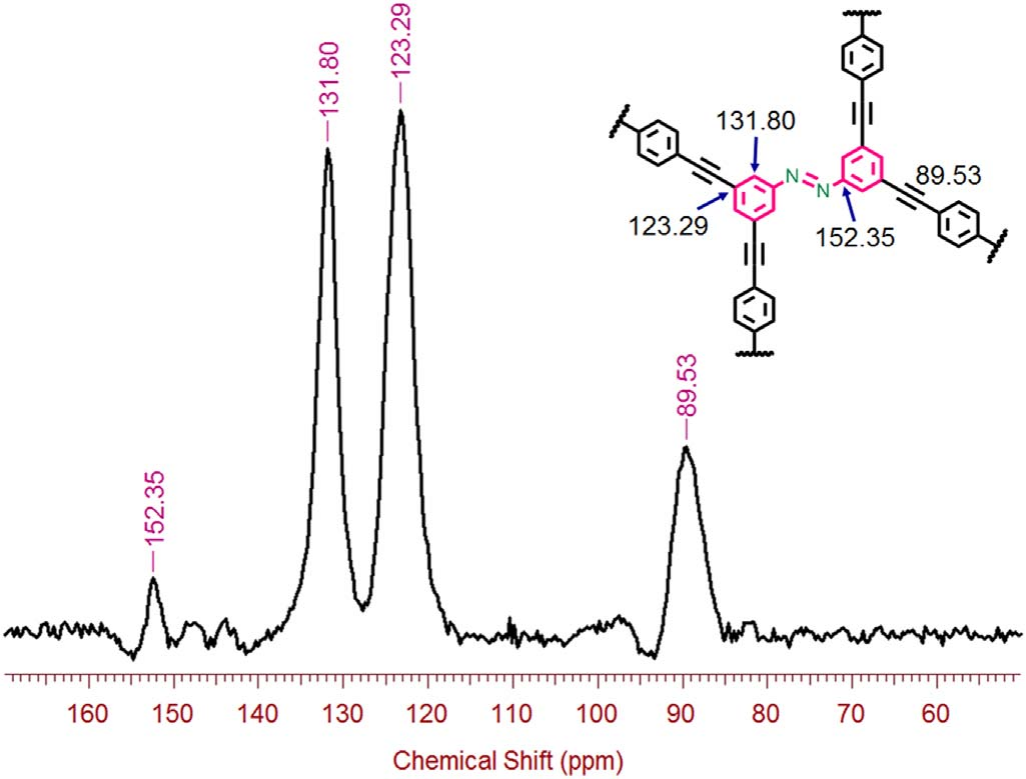
Solid-state ^13^C-CP/MAS spectrum of Azo–POP. Chemical structure of the repeating unit highlighting different C-atoms has been given for easy comparison.

**Fig. 2:**
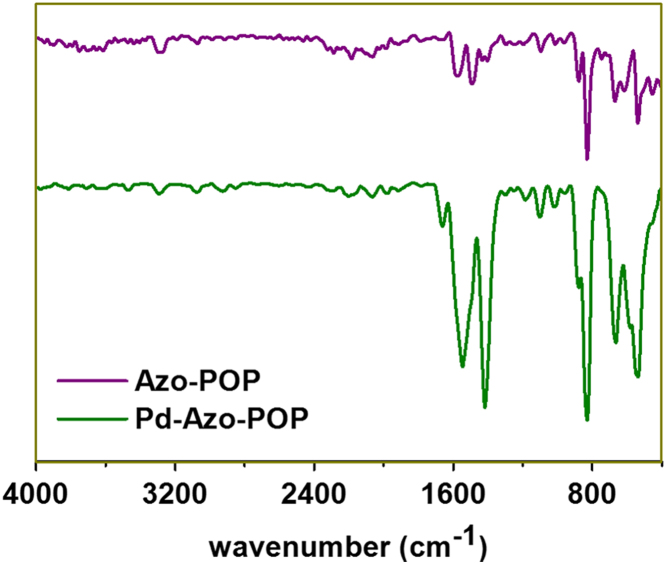
FT-IR spectra (neat) of Azo–POP and Pd–Azo–POP.

**Fig. 3:**
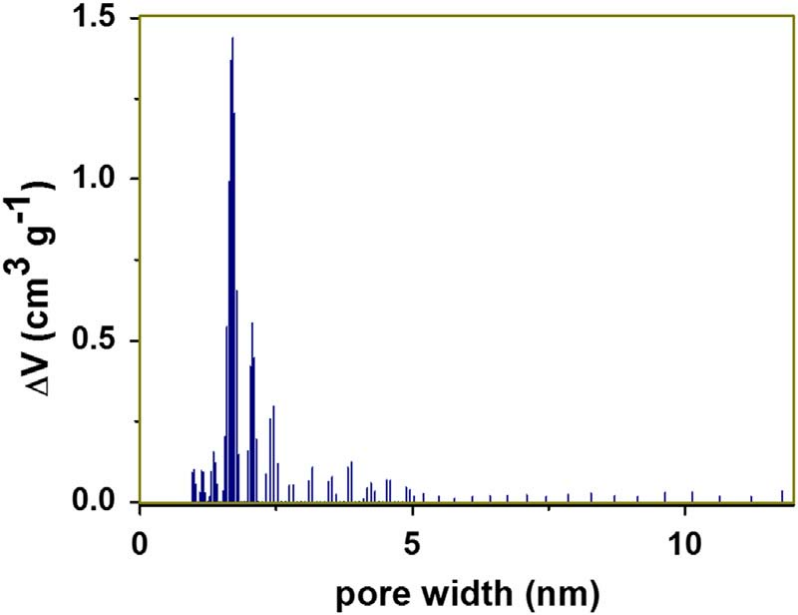
Pore size distribution of Azo–POP showing two maxima at 1.71 and 3.86 nm.

**Fig. 4:**
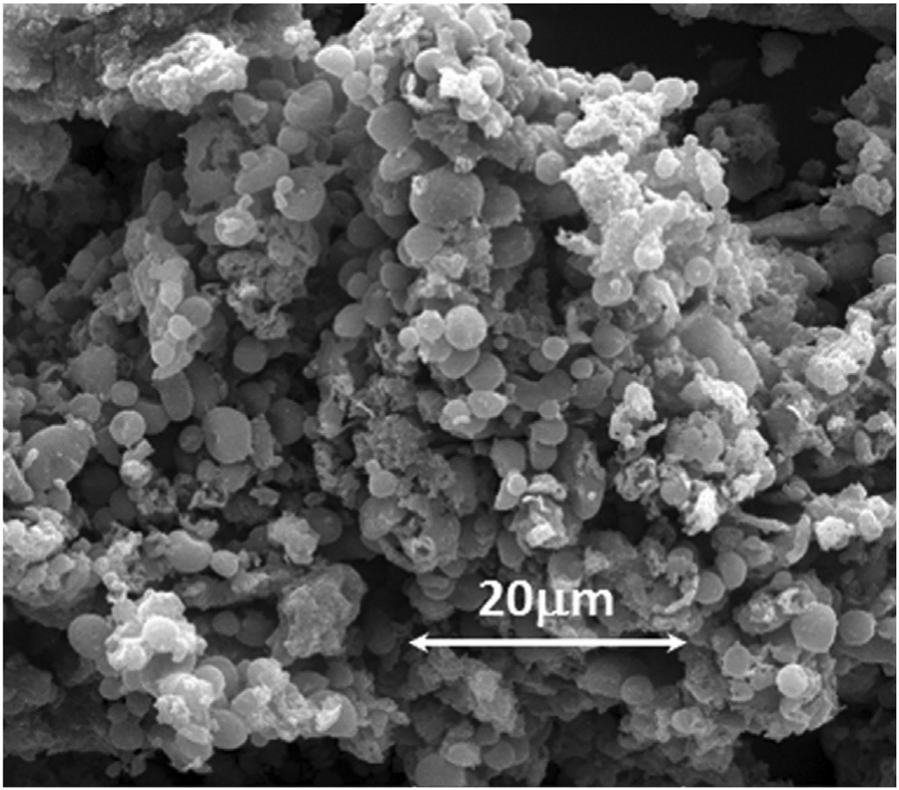
FESEM image of Pd–Azo–POP.

**Fig. 5:**
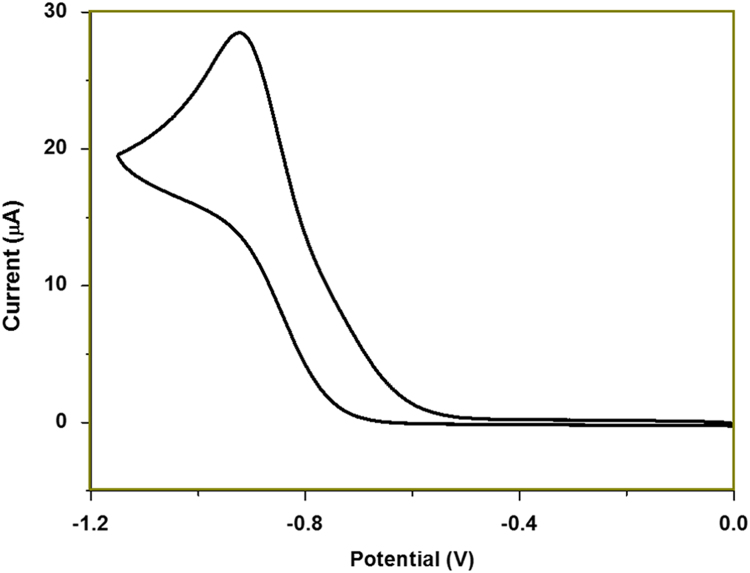
Cyclic voltammogram analysis of Azo–POP. (CH_2_Cl_2._ Scan rate 50 mV s^−1^).

**Fig. 6:**
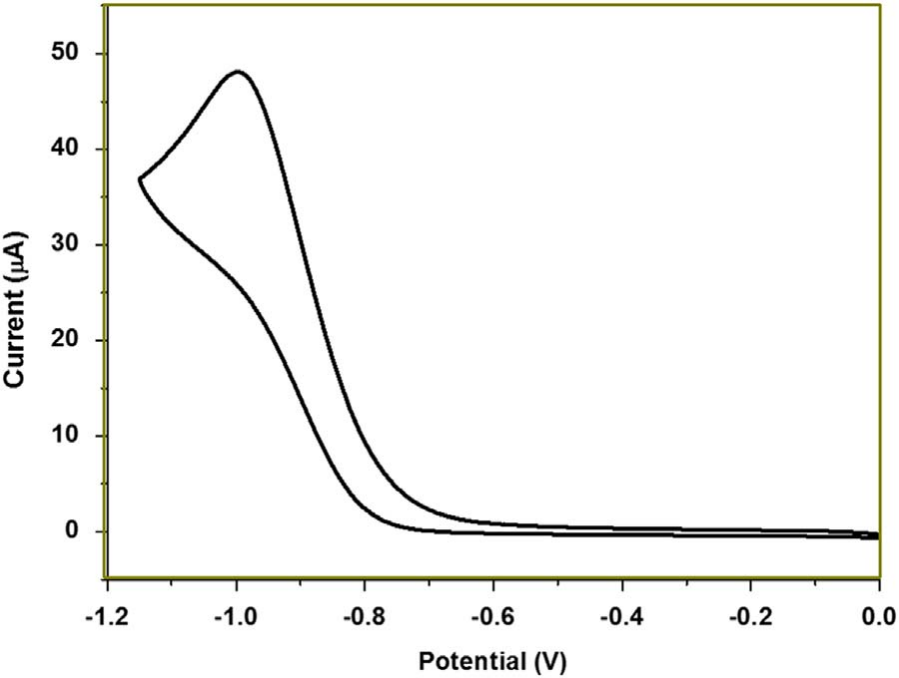
Cyclic voltammogram analysis of Pd–Azo–POP. (CH_2_Cl_2._ Scan rate 50 mV s^−1^).

**Fig. 7:**
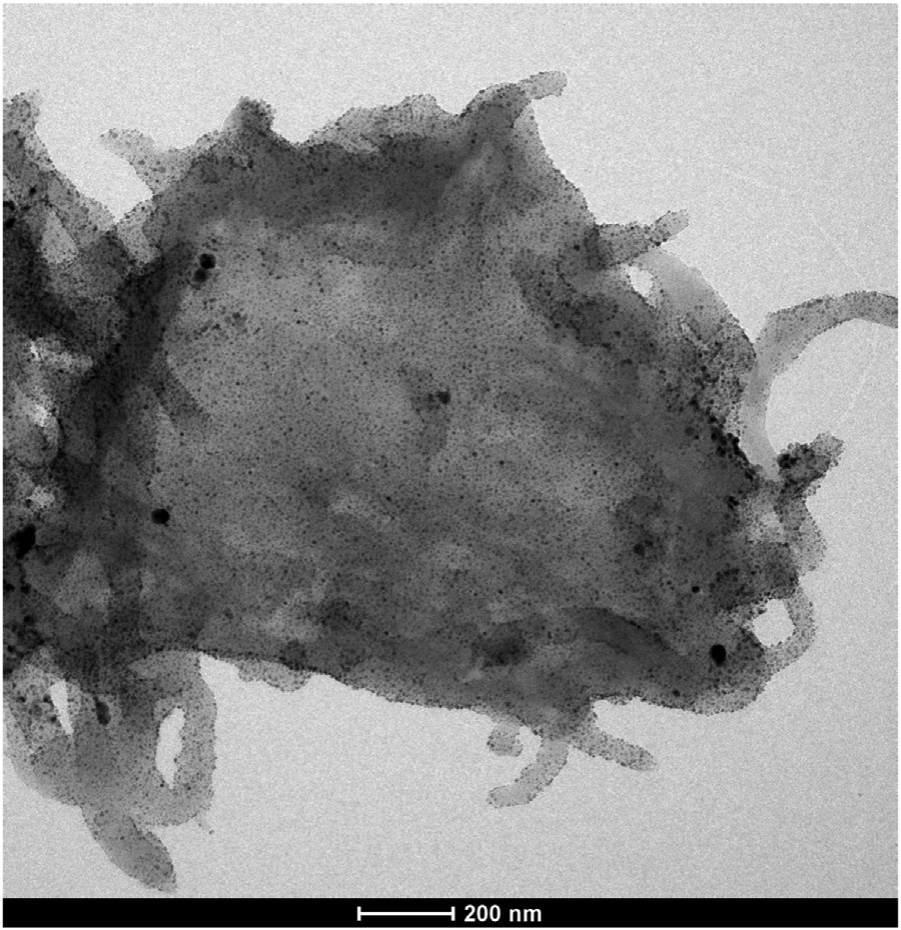
TEM image of Pd–Azo–POP after recycling tests.

**Fig. 8:**
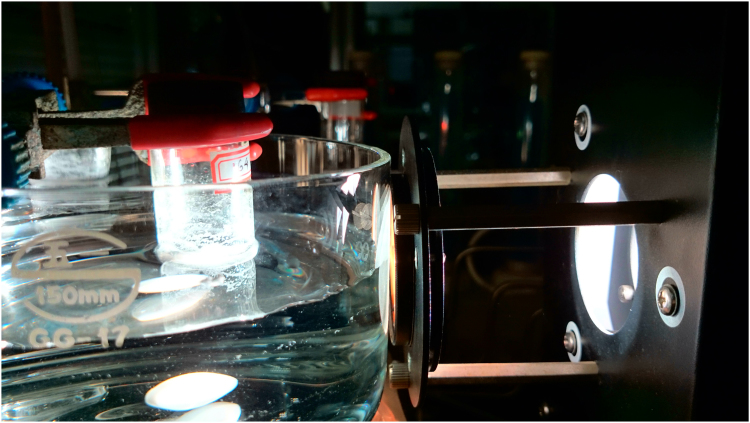
Digital image of the photocatalytic set-up while Suzuki coupling reaction in progress.

**Fig. 9:**
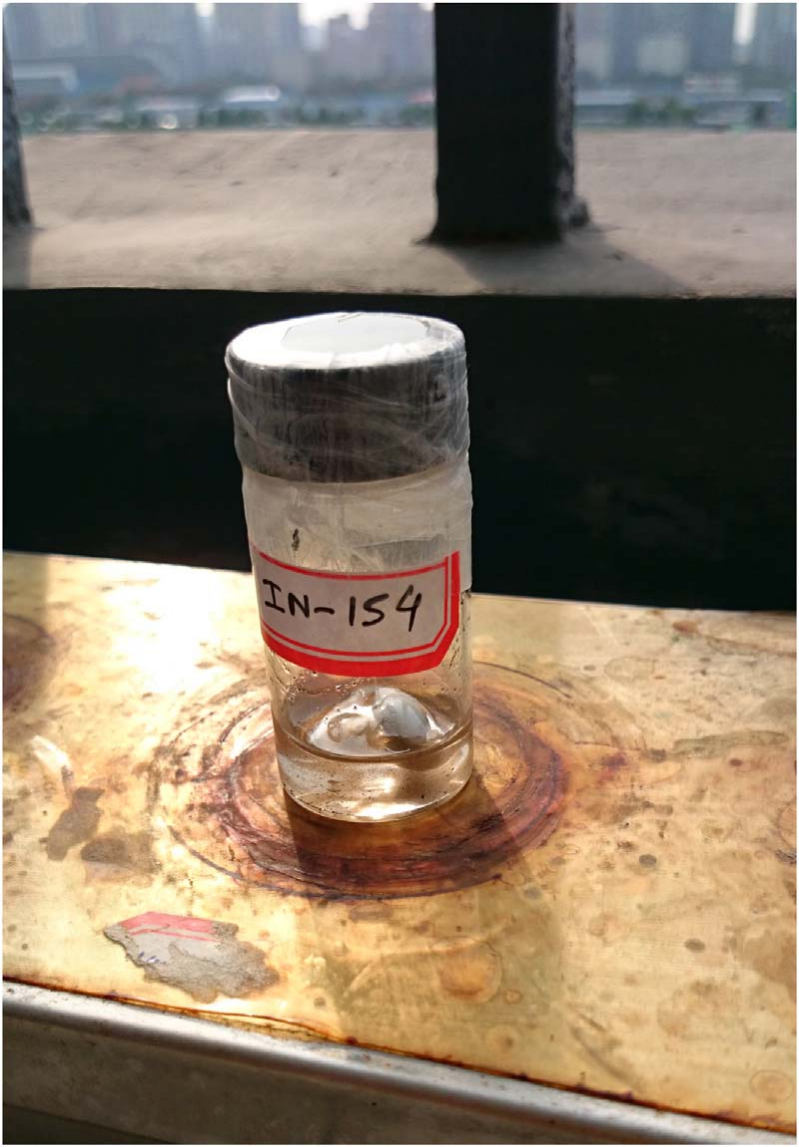
Digital image of natural sunlight-induced coupling reaction in progress.

**Fig. 10:**
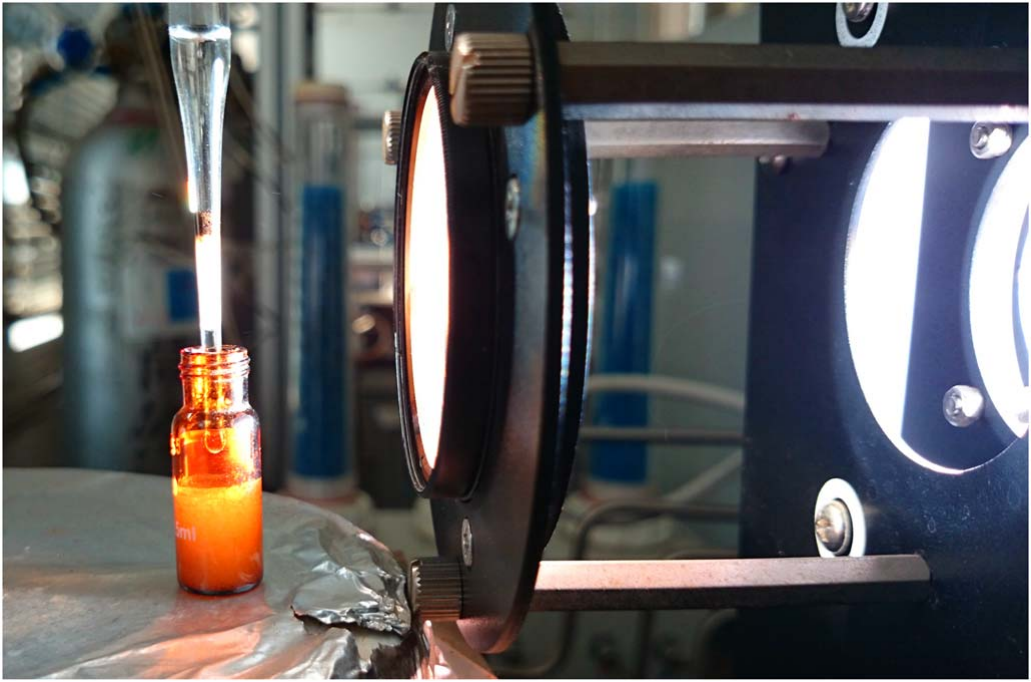
Digital image of continuous flow set-up. Instantaneous appearance of the white crystalline product can be seen.

**Scheme 1:**
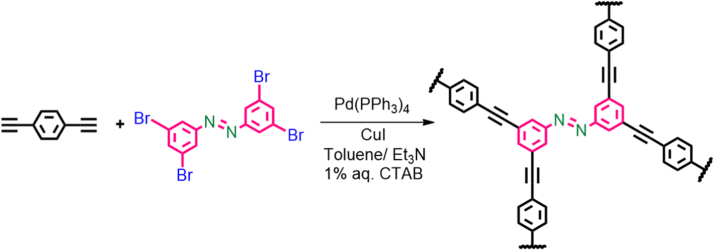
Synthetic route to Azo–POP starting from respective monomer and co-monomer.

Fig. 1, Fig. 3 demonstrate solid-state ^13^C-CP/MAS NMR spectra and pore size distribution pattern of Azo–POP respectively, while Fig. 2 shows a comparative FT-IR pattern of Azo–POP and Pd–Azo–POP. FESEM image of Pd–Azo–POP is given in Fig. 4. Fig. 5, Fig. 6 demonstrate the cyclic voltammogram analysis of the materials and Fig. 7 shows the TEM image of Pd–Azo–POP after recycling. Digital images of the photocatalytic setup under visible light, natural sunlight, and prototypal continuous flow method are given in Fig. 8, Fig. 9, Fig. 10, respectively. The synthesis of Azo–POP is illustrated in Scheme 1. Table 1 presents the screening conditions for visible light-induced Suzuki coupling, whereas Table 2 demonstrates the data for controlled experiments performed to propose a photocatalytic Suzuki mechanism. ^1^H and ^13^C solution state NMR data for the catalytic Suzuki products are given as text in “NMR data of the catalytic products” section.

**Table 1:** Different screening conditions and corresponding yield for visible light-induced Suzuki coupling of iodobenzene and phenylboronic acid^a^.

Entry	Base^b^	Solvent	Time (h)	Yield (%)^d^
1	K_2_CO_3_	DMF	96	42
2	Cs_2_CO_3_	DMF	96	73
3	Cs_2_CO_3_	DMF	120	94
4	Cs_2_CO_3_	DMF/water^e^	24	100
5	Cs_2_CO_3_	Water	8	71
6	Cs_2_CO_3_	Water^c^	4	100
7	Cs_2_CO_3_	EtOH	5.5	98
8	Cs_2_CO_3_	EtOH /water^e^	4	100

a Reaction conditions: Iodobenzene, 0.5 mmol; phenylboronic acid, 0.55 mmol; base; Pd–Azo–POP, 200 μL; solvent, 3 mL; reaction temperature, 25 °C; water bath; PL-XQ 350 W Xe lamp with 420 nm UV cut-off.

b Base amounts of entry 1 and 2, 1 mmol; base amounts of entry 3–8, 0.75 mmol.

c CTAB, 0.5 mmol.

d Isolated yields after chromatography.

e 1:1 v/v mixture.

**Table 2:** Control reactions between p-methoxyiodobenzene and phenylboronic acid under different conditions and corresponding product yields for mechanistic investigation.

Entry	Conditions	Time (h)	Yield^b^
1	Standard^a^	4	>99
2	In dark	8	7
3	Without base^c^	4	—
4	Azo–POP without Pd^c^	12	trace
5	Pd/C as catalyst^c^^,^^d^	4	8
6	Hole scavenger (EDTA)^c^	4	—e
7	Electron scavenger (BQ)^c^	4	13^f^

a Standard reaction condition: p-methoxyiodobenzene, 0.5 mmol; phenylboronic acid, 0.55 mmol; Cs_2_CO_3_, 0.75 mmol; Pd–Azo–POP, 200 μL; 1:1 ethanol/water, 3 mL; reaction temperature, 25 °C; water bath; PL-XQ 350 W Xe lamp with 420 nm UV cut-off.

b Isolated yields after chromatography.

c Visible light.

d 10 wt% Pd/C, 3.2 mg.

e 17% yield of 4,4′-dimethoxy-1,1′-biphenyl Ullmann product.

f 6% yield of 1,1′-biphenyl by-product.

## 2 Experimental design, materials, and methods

### 2.1 Materials

The materials used for synthesizing the polymer network were purchased from Aladdin (China). All reagents for the photocatalytic Suzuki coupling reaction were purchased from TCI (Japan), Aladdin (China), and J&K Chemicals (China). All the solvents (AR grade) were obtained from Aladdin (China) and used without further purification.

### 2.2 Methods

Solution-state NMR spectra were recorded on a Bruker 500 MHz NMR spectrometer (^1^H NMR, 500 MHz; ^13^C NMR, 126 MHz). The peak frequencies were referenced with respect to TMS (*δ* = 0 ppm) as an internal standard for ^1^H NMR, and against the solvent (CDCl_3_, *δ* = 77 ppm) for ^13^C NMR, respectively. The coupling constants, J, were reported in Hz. The Pd-content in Pd–Azo–POP was determined by inductively coupled plasma optical emission spectra (ICP, Varian VISTAMPX). All other characterizations of the materials including solid-state ^13^C-CP/MAS NMR, FT-IR, powder XRD, porosity, and adsorption–desorption isotherms, Scanning Electron Microscopy, Transmission Electron Microscopy, X-ray Photoelectron Spectroscopy, UV–vis absorptions, Electron Paramagnetic Resonance measurements, and Cyclic Voltammogram were performed following the previously reported method [2].

### 2.3 Experimental design

For detailed experimental design procedures containing material synthesis, and catalytic setup in batch mode as well as in continuous flow technique please see associated research article [1].

### 2.4 NMR data of the catalytic products

**1,1׳-Biphenyl**.

^1^H NMR (CDCl_3_, 500 MHz, TMS, ppm): δ 7.38 (t, *J*= 7.3 Hz, 2 H), 7.47 (t, *J*= 7.7 Hz, 4 H), 7.63 (d, *J*= 7.5 Hz, 4 H). ^13^C NMR (CDCl_3_, 126 MHz, ppm): δ 127.22, 127.30, 128.80, 141.31.

**4-Methyl-1,1׳-biphenyl** [3].

^1^H NMR (CDCl_3_, 500 MHz, TMS, ppm): δ 2.43 (s, 3 H), 7.28 (d, *J*= 7.9 Hz, 2 H), 7.35 (t, *J*= 7.4 Hz, 1 H), 7.46 (t, *J*= 7.7 Hz, 2 H), 7.53 (d, *J*= 8 Hz, 2 H), 7.61 (d, *J*= 7.9 Hz, 2 H). ^13^C NMR (CDCl_3_, 126 MHz, ppm): δ 21.15, 127.02, 128.76, 129.53, 131.25, 137.05, 137.29, 138.42, 141.22.

**4-Methoxy-1,1׳-biphenyl** [3].

^1^H NMR (CDCl_3_, 500 MHz, TMS, ppm): δ 3.86 (s, 3 H), 6.99 (d, *J*= 8.8 Hz, 2 H), 7.31 (t, *J*= 7.3 Hz, 1 H), 7.42 (t, *J*= 7.7 Hz, 2 H), 7.54 (d, *J*= 8.9 Hz, 2 H), 7.56 (d, *J*= 7.9 Hz, 2 H). ^13^C NMR (CDCl_3_, 126 MHz, ppm): δ 55.37, 114.23, 126.67, 126.76, 128.18, 128.74, 133.82, 140.86, 159.18.

**4-Hydroxy-1,1׳-biphenyl** [3].

^1^H NMR (CDCl_3_, 500 MHz, TMS, ppm): δ 6.96 (d, *J*= 8.5 Hz, 2 H), 7.30 (t, *J*= 7.3 Hz, 1 H), 7.41 (t, *J*= 7.6 Hz, 2 H), 7.47 (d, *J*= 8.5 Hz, 2 H), 7.55 (d, *J*= 7.9 Hz, 2 H). ^13^C NMR (CDCl_3_, 126 MHz, ppm): δ 115.78, 126.65, 128.22, 128.67, 133.09, 133.93, 141.06, 156.28.

**4-Fluoro-1,1׳-biphenyl** [3].

^1^H NMR (CDCl_3_, 500 MHz, TMS, ppm): δ 7.16 (t, *J*= 8.7 Hz, 2 H), 7.38 (t, *J*= 7.4 Hz, 1 H), 7.47 (t, *J*= 7.6 Hz, 2 H), 7.58 (dd, *J*= 7.9, 4.2 Hz, 4 H). ^13^C NMR (CDCl_3_, 126 MHz, ppm): δ 115.54, 115.71, 127.04, 127.27, 128.67, 128.74, 128.84, 137.36, 137.39, 140.29, 161.52, 163.48.

**Methyl-4-phenylbenzoate**.

^1^H NMR (CDCl_3_, 500 MHz, TMS, ppm): δ 3.94 (s, 3 H), 7.39 (t, *J*= 8.5 Hz, 1 H), 7.47 (t, *J*= 7.5 Hz, 2 H), 7.63 (d, *J*= 8.0 Hz, 2 H), 7.67 (d, *J*= 8.1 Hz, 2 H), 8.11 (d, *J*= 8.1 Hz, 2 H). ^13^C NMR (CDCl_3_, 126 MHz, ppm): δ 53.42, 127.04, 127.26, 128.13, 128.83, 128.91, 130.08, 139.90, 145.65, 166.89.

**4-Cyano-1,1׳-biphenyl** [3].

^1^H NMR (CDCl_3_, 500 MHz, TMS, ppm): δ 7.42 (t, *J*= 7.2 Hz, 1 H), 7.48 (t, *J*= 7.6 Hz, 2 H), 7.59 (d, *J*= 7.9 Hz, 2 H), 7.69 (d, *J*= 8.2 Hz, 2 H), 7.73 (d, *J*= 8.3 Hz, 2 H). ^13^C NMR (CDCl_3_, 126 MHz, ppm): δ 110.90, 118.90, 127.21, 127.73, 128.65, 129.09, 132.57, 139.16, 145.69.

**1,4-Biphenylbenzene** [3].

^1^H NMR (CDCl_3_, 500 MHz, TMS, ppm): δ 7.36 (t, *J*= 7.3 Hz, 2 H), 7.46 (t, *J*= 7.6 Hz, 4 H), 7.64 (d, *J*= 7.9 Hz, 4 H), 7.68 (s, 4 H). ^13^C NMR (CDCl_3_, 126 MHz, ppm): δ 127.08, 127.37, 127.53, 128.84, 140.16, 140.74.

**1-Phenylnaphthalene**.

^1^H NMR (CDCl_3_, 500 MHz, TMS, ppm): δ 7.49-7.33 (m, 9 H), 7.89-7.79 (m, 3 H). ^13^C NMR (CDCl_3_, 126 MHz, ppm): δ 125.50, 126.02, 126.20, 127.12, 127.34, 127.81, 128.32, 130.07, 131.64, 133.87, 140.27, 140.77.

**2, 6-Biphenylpyridine**.

^1^H NMR (CDCl_3_, 500 MHz, TMS, ppm): δ 7.49 (t, *J*= 7.1 Hz, 2 H), 7.55 (t, *J*= 7.3 Hz, 4 H), 7.63 (d, *J*= 7.8 Hz, 2 H), 8.02 (d, *J*= 7.9 Hz, 1 H), 8.28 (d, *J*= 7.5 Hz, 4 H). ^13^C NMR (CDCl_3_, 126 MHz, ppm): δ 119.00, 127.06, 128.81, 129.64, 137.66, 140.08, 158.63.

**4-Carbaldehyde-1,1׳-biphenyl**.

^1^H NMR (CDCl_3_, 500 MHz, TMS, ppm): δ 7.45 (t, *J*= 7.2 Hz, 1 H), 7.51 (t, *J*= 7.5 Hz, 2 H), 7.67 (d, *J*= 7.6 Hz, 2 H), 7.78 (d, *J*= 7.9 Hz, 2 H), 7.98 (d, *J*= 7.9 Hz, 2 H), 10.09 (s, 1 H). ^13^C NMR (CDCl_3_, 126 MHz, ppm): δ 127.38, 127.70, 129.03, 130.28, 135.23, 139.74, 147.23, 191.94.

**Diphenylmethane**.

^1^H NMR (CDCl_3_, 500 MHz, TMS, ppm): δ 4.00 (s, 2 H), 7.16–7.23 (m, 4 H), 7.24–7.32 (m, 6 H). ^13^C NMR (CDCl_3_, 126 MHz, ppm): δ 41.99, 126.11, 128.51, 129.04, 141.19.

**Benzophenone**.

^1^H NMR (CDCl_3_, 500 MHz, TMS, ppm): δ 7.46–7.58 (m, 4 H), 7.59–7.64 (m, 2 H), 7.84 (d, *J*= 7.6 Hz, 4 H). ^13^C NMR (CDCl_3_, 126 MHz, ppm): δ 128.27, 130.02, 132.38, 137.61, 196.68.
